# Why concerns about vitamin D deficiency should not lead to over testing and overtreatment

**DOI:** 10.1080/13814788.2020.1850019

**Published:** 2020-12-09

**Authors:** Cristina Carbonell-Abella

**Affiliations:** Group on Study of Osteoporosis, Spanish Society of Family Medicine, University of Barcelona, Via Roma Health Centre, Barcelona, Spain

In recent years, there has been a growing interest in vitamin D with a significant increase in the number of measurements worldwide [1]. However, there are controversies about the necessity of these determinations and on the cut-off point defining vitamin D deficiency. It is possible that a single cut-off point cannot be established and different scenarios might be assessed, such as those represented by patients with osteoporosis, kidney failure, diabetes or other circumstances, and the healthy asymptomatic population.

In their article published in this issue of the journal, Göktas et al. estimate the prevalence of hypovitaminosis D based on concentrations of 25-OH vitamin D in a large sample of adult demanding population attending health centres in Bursa [2], Turkey. The mean age of the 11,734 patients was 46.5 ± 16.9 years (45.4 ± 16.4 years in women and 51.1 ± 17.9 years in men). The mean concentration in the entire population was 16.6 ± 11.5 ng/ml, (15.8 ± 11.8 ng/ml in women and 19.6 ± 10 ng/ml in men). By using the cut-off point for the general population of 30 ng/ml, the authors consider that 70.6% of the population is deficient in vitamin D, 19.5% insufficient and only 9.9% have normal concentrations. The data are interesting, but what is the clinical significance of these data; are they useful for making clinical decisions? We would like to have the figures stratified by age groups, and by different clinical situations.

The authors discuss that unlike what is reported in the literature, they found higher values of 25-OH vitamin D among patients with type 2 diabetes mellitus, hypertension, dyslipidaemia and cardiovascular disease. The article suggests the need to promote population screening to correct supposed deficits.

## What does vitamin D do?

Vitamin D has a crucial role in bone metabolism [3]. It maintains the phosphocalcic balance and regulates bone remodelling. It is considered that an adequate concentration is necessary to optimise the effectiveness of the different therapies for osteoporosis. The classic consequences of sustained and severe vitamin D deficiency are rickets in children and osteomalacia in adults, but it is also ascribed a role in osteoporosis [4]. The results of vitamin D in the prevention of fractures, prevention of falls, and effects on muscles are diverse. This diversity depends on the characteristics of the patients included in the studies, the baseline values of 25-OH vitamin D, and other characteristics [5,6]. The benefit of taking vitamin D is more significant among those with low values (<30 nmol/L; <12 ng/ml) and a high risk of falls or fractures [7].

On the other hand, low concentrations of vitamin D have been associated with a wide variety of nonskeletal processes and diseases, such as cardiovascular disease, high blood pressure, diabetes, immune disorders, cancer or depression [8]. All this information is drawn from observational studies.

However, prospective placebo-controlled studies that should corroborate the efficacy of increasing these concentrations show inconclusive results [9]. A recent meta-analysis, which reviews the evidence from the latest publications from 2012 to 2017, does find benefit from vitamin D supplementation in respiratory tract infections, asthma exacerbations, and cancer mortality [10]. Although the final 25-OH vitamin D concentrations are higher than in previous studies, they find no benefit from supplementation for conditions such as cardiovascular disease, glucose metabolism, muscle function, or colorectal adenoma.

## What to know about laboratory testing for vitamin D

The interpretation of vitamin D results should be considered according to the clinical situation and bearing in mind the variability of the technique used for its determination. There are different methods, with the most widely used being the competitive immunoassay and liquid chromatography coupled with tandem mass spectrometry [11,12]. Most of the studies carried out over the last 20–30 years have used the immunoassay technique, and even today, it is the technique used in many laboratories. Its main limitation is the imprecision and variability, which can reach 15–20%. These techniques frequently underestimate or overestimate vitamin D concentrations at the upper and lower end of their measurement range, where precision would be most important. Liquid chromatography coupled with tandem mass spectrometry is currently considered the gold standard, due to its greater precision, specificity, sensitivity and reproducibility. The vitamin D standardisation program specifies that 10% is an acceptable variation in precision. In practice, this level of precision indicates that, if the determination value is 30 ng/ml, considering 95% of certainty, we can ensure that the value will be between 24–36 ng/ml (60–90 nmol/L).

The plasma concentration of 25-OH vitamin D shows significant intra-individual and inter-individual variability. This variability is influenced by the season of the year, genetic factors, age, body mass index, skin pigmentation, sun exposure, and diet. These factors, and specifically the seasonal variation, should always be considered when requesting a serum 25-OH vitamin D determination and interpreting the results.

## What is an optimal vitamin D value?

There is controversy about the optimal values of vitamin D. Various organisations and scientific societies use different definitions for deficiency, insufficiency or optimal values of vitamin D. The American Institute of Medicine (IOM) formulated its recommendations in 2011, defining deficiency when the concentration of vitamin D is less than 12 ng/ml (30 nmol/L), insufficiency between 12–20 ng/ml (30–50 nmol/L) and appropriate values above 20 ng/ml (>50 nmol/L) [13]. Other organisations also support this recommendation. The American Endocrinology Society issued its recommendations also in 2011, considering concentrations <20 ng/ml (<50 nmol/L) deficient, between 20–30 ng/ml (52.5 − 72.5 nmol/L) insufficient, and above 30 ng/ml (>77.5 nmol/L) adequate [14].

Both recommendations agree that values below 10–12 ng/ml (25–30 nmol/L) reflect severe deficiency and are associated with an increased risk of rickets, osteomalacia, or diffuse pain. For the general population, concentrations >20 ng/ml probably are sufficient. According to the IOM, for 97.5% of the American population and in patients with pathology of bone metabolism, digestive disorders, kidney or other objectives, concentrations above 30 ng/ml will be optimal.

## No screening for vitamin D deficiency

There is currently no evidence to demonstrate the benefits of vitamin D deficiency screening in the general population [15]. It would be necessary to demonstrate feasibility, cost-effectiveness and benefits in terms of health. In the absence of this evidence, screening is not recommended in subjects who are not at risk. The determination is recommended only in subjects with symptoms (weakness, muscle pain, generalised bone pain) or signs of deficiency and in patients at risk of suffering from it and in whom a rapid response is expected after optimising the concentration of 25-OH vitamin D.

## Testing for and supplementing vitamin D in individual patients

Although there is consensus on not doing population screening, there is no unanimity about when vitamin D measurement is indicated, as this ranges from very restrictive to practically everyone. Most of the recommendations are expert judgement with little scientific evidence. 25-OH Vitamin D should be determined in patients with bone metabolic pathology, patients with malabsorptive syndromes such as cystic fibrosis, coeliac disease, Crohn's disease, gastric bypass, some medications such as orlistat, patients with obesity, liver or kidney failure, granulomatous diseases, hyper or hypoparathyroidism, hypocalcaemia, hypercalcaemia and those who receive some treatments such as glucocorticoids, anticonvulsants or HAART therapy. It should also be determined when toxicity is suspected in patients taking high doses of vitamin D for long periods, with laboratory abnormalities or associated characteristic symptoms.

Vitamin D needs vary from person to person. The average daily need is estimated at 400 IU/day. The recommended intake is 600 IU/day for adults up to 70 years and 800 IU/day for those over 70 years. It is recommended not to exceed the tolerable upper limit, which would be 4,000 IU/day (IOM) for adults and the elderly. However, some studies have tested higher doses, up to 10,000 or more units per day without showing a risk. Obtaining enough vitamin D from natural sources can be difficult for certain population groups. Enriched food and sufficient sun exposure are essential to achieve correct vitamin D status. For every 2.5 µg (100 IU)/day the 25 OH-vitamin D increases between 2.5–5 nmol/L (0.5–1 ng/ml), but huge variability is described.

The prescription of supplements to the general population is not recommended, since treating asymptomatic subjects without belonging to risk groups has not been shown to improve health. Some organisations routinely recommend supplementing high-risk groups, without the need to previously determine or monitor 25-OH vitamin D concentrations, such as patients with dark complexions (African, Central American and South-West Asian), institutionalised patients, at high risk of falls or fragility fractures, subjects with little or no solar exposure for cultural, medical or other reasons, children under one year of age with exclusive breastfeeding, all children between 1–4 years and also children and adults with obesity (BMI >30). The recommended dose is 400 IU (10 µg)/day, which would prevent rickets and osteomalacia and is unlikely to have harmful effects.

## Conclusion

We can conclude that in recent years there has been a growing interest in the possible deficiency of vitamin D and the associated clinical effects. This has led to an exponential increase in the number of determinations, followed by treatments and re-tests that are not always appropriate. Applications must be adapted to groups at particular risk, in which it is essential to confirm the deficiency and correct it promptly.
